# Automatic Leaf Segmentation for Estimating Leaf Area and Leaf Inclination Angle in 3D Plant Images

**DOI:** 10.3390/s18103576

**Published:** 2018-10-22

**Authors:** Kenta Itakura, Fumiki Hosoi

**Affiliations:** Graduate School, University of Tokyo, Tokyo 113-8657, Japan; itakura-kenta095@g.ecc.u-tokyo.ac.jp

**Keywords:** leaf segmentation, three-dimensional imaging, leaf inclination angle estimation, leaf area estimationutf8

## Abstract

Automatic and efficient plant monitoring offers accurate plant management. Construction of three-dimensional (3D) models of plants and acquisition of their spatial information is an effective method for obtaining plant structural parameters. Here, 3D images of leaves constructed with multiple scenes taken from different positions were segmented automatically for the automatic retrieval of leaf areas and inclination angles. First, for the initial segmentation, leave images were viewed from the top, then leaves in the top-view images were segmented using distance transform and the watershed algorithm. Next, the images of leaves after the initial segmentation were reduced by 90%, and the seed regions for each leaf were produced. The seed region was re-projected onto the 3D images, and each leaf was segmented by expanding the seed region with the 3D information. After leaf segmentation, the leaf area of each leaf and its inclination angle were estimated accurately via a voxel-based calculation. As a result, leaf area and leaf inclination angle were estimated accurately after automatic leaf segmentation. This method for automatic plant structure analysis allows accurate and efficient plant breeding and growth management.

## 1. Introduction

In the growth and developmental processes, plant structures change during the seasons because of environmental processes [1]. For accurate plant breeding and growth monitoring, it is necessary to obtain information about the plant structures affecting the plant status [2,3,4]. For example, leaf area index (LAI) is an important plant structural parameter because it determines the primary photosynthetic production, plant evaporation, and plant growth characterization [5]. Leaf inclination angle can also be an informative plant structural parameter because it can be an indicator of a growth-state, such as a water condition [6]. It relates greatly to increasing photosynthetic productivity [7]. Thus, LAI and leaf inclination angles are important plant structural parameters. However, the conventional methods of measuring LAI and leaf inclination angles are destructive and tedious [5,8]. 

Two-dimensional (2D) imaging techniques have been utilized for obtaining structural parameters, such as height, stem diameter, and leaf area, which have led to plant functional analysis. However, techniques based on 2D imaging are insufficient for investigating the spatial plant structures because plants have complicated three-dimensional (3D) structures [4,9,10,11]. Moreover, because a 2D image is essentially a projected image in one direction, some leaves in 2D images are hidden by others (occlusion), and the structural parameters are difficult to estimate accurately. Thus, 2D projections remove important parts of the information and fail to exploit the full potential of shape analysis [11]. Thus, construction of 3D models of plants and acquisition of their spatial information is an effective method of overcoming these problems. This leads to plant functions and conditions necessary for appropriate breeding and growth management. 

For 3D model construction, lidar (light detection and ranging) currently attracts attention. It is a 3D scanner that measures the distance to a target using the elapsed time between the emission and return of laser pulses. It can record many 3D point-cloud data of a target, from which information about plant structural parameters, such as shape, LAI, leaf area density, leaf inclination angle, location, height, and volume, can be extracted [12,13,14,15,16,17,18,19,20]. Another method is the stereo vision system, which constructs 3D point clouds from a set of two images [21,22]. Takizawa et al. [23] used stereo vision to construct plant 3D models, and Mizuno et al. [24] used stereo vision for wilt detection. With the exponential increase in computational power and the widespread availability of digital cameras, the use of a photogrammetric approach (i.e., structure from motion (SfM)) has generated 3D point-cloud models from 2D imagery and has become widespread [1,25,26,27,28,29]. The SfM approach requires multiple images of a scene taken from different positions. From those images, camera parameters and camera positions are calculated, and 3D point-cloud images are constructed [30]. More detailed 3D models can be constructed by this method, compared to stereo vision, owing to the use of multiple scene images. In previous studies, plant shape (e.g., height, stem length, leaf area) could be estimated accurately from 3D models constructed by SfM [4,28]. 

After obtaining detailed 3D plant structural models, automatic detection of individual leaves becomes a fundamental and challenging task in agricultural practices [31]. For example, in phenotyping, to reduce labor cost, there has been a growing interest in developing solutions for the automated analysis of visually observable plant traits [32]. To extract plant traits, automatic leaf segmentation in 3D images is essential. If leaves in 3D images are segmented automatically, it becomes possible to automatically extract structural plant traits. Furthermore, automatic segmentation can be applied to many related works in agricultural automation (e.g., de-leafing, plant inspection, pest management [31]).

In previous studies on 3D segmentation method, Alenya et al. [33] and Xia et al. [31] proposed segmentation methods using depth sensors. However, the methods had problems with low segmentation accuracy and difficulty under the condition that neighboring leaves were near one another. Paproki et al. [10] presented a 3D mesh-based technique developed for leaf segmentation. In that study, 3D point-cloud models were converted into mesh-based 3D models, and tubular fittings were implemented to organs as stems or petioles. Afterwards, each leaf of a 3D image was segmented [34]. However, when mesh-based 3D models were built, all regions in the 3D model could not be filled with meshes. Thus, some holes occurred on the surfaces. Because this segmentation method utilized the normal vector of each polygon, and whole-leaf surfaces had to be filled with meshes, the holes on the 3D model had to be filled manually, so that the entire segmentation process is difficult to fully automate. The segmentation of 3D leaves, which uses top-view 2D images, were also advocated by Kaminuma et al. [35] and Teng et al. [36]. In these studies, k-curvature and graph-cut algorithms were used for leaf segmentation. Those methods had difficulty segmenting overlapped leaves in the top view. Thus, their application was limited to simple plants having only a few leaves without overlapping in the top-view images.

Moreover, estimation of automatic plant structures, such as leaf area and leaf inclination angle, were also difficult. In the case of mesh-based 3D modeling, leaf area and leaf inclination angle could be calculated based on the area and normal vector of each polygon mesh. However, the construction of mesh-based 3D models entailed manual operations to fill holes on the surface of leaves. Thus, the automatic extraction of the plant structure parameters was difficult.

In previous segmentation methods, accuracy was not satisfactory and the samples were limited to those having few leaves; the fully automatic process remained difficult. Furthermore, automatic estimation of structural parameters of plants was also difficult. 

In this study, we propose methods for accurate and automatic leaf segmentation and retrieval of plant structural parameters using 3D point-cloud images, overcoming disadvantages of previous studies. Leaves in 3D models were segmented automatically with a 3D point-cloud processing method combined with a 2D image processing method. After the process, leaf area and leaf inclination angle in each segmented leaf were estimated accurately and automatically.

## 2. Materials and Methods

### 2.1. Plant Material

For the experiments, we selected small plants (i.e., Pothos (*Epipremnum aureum*), Hydrangea (*Hydrangea macrophylla*), Dwarf schefflera (*Schefflera arboricola*), Council tree (*Ficus altissima*), Kangaroo vine (*Cissus antarctica*), Umbellata (*Ficus umbellate*), and Japanese sarcandra (*Sarcandra glabra*)). The height and number of leaves ranged from 20 to 60 cm and from 3 to 11, respectively. 

### 2.2. 3D Reconstruction of Plants

The camera used in image acquisition for 3D reconstruction was a Canon EOS M2 (Canon Inc., Tokyo, Japan). The camera was hand-held, and about 80 images were recorded for each sample. The number of images was set to obtain sufficient 3D resolution for each leaf to be clearly observed. For 3D reconstruction image acquisition, we referred to Rose et al. [28], where images were taken by moving around the sample and closing the circle. The distance from the camera to the plant was about 30–100 cm. The images were taken from an oblique angle or horizontally. The resolution of the image was 3456 × 5184 pixels. For 3D point-cloud image construction using SfM [37], camera calibration was done using Agisoft Lens (Agisoft LCC, Saint Petersburg, Russia). For the calibration, about 100 images of a checkerboard were taken from various angles and positions, and in the software, image corners were extracted automatically and the parameters for the calibration were calculated. Then, using the software Agisoft Photoscan Professional (Agisoft LCC, Russia), plant 3D point-cloud images were reconstructed [38]. The reconstructed models did not have any spatial scale and their coordinates did not correspond to world coordinates. Thus, a cube-shaped reference, such as a box (13×12×9), was placed around the plant, and the scale and coordinate of the 3D image was determined [38].

### 2.3. Automatic Leaf Segmentation and Its Evaluation

#### 2.3.1. Conversion into Voxel Coordinate

In the reconstructed 3D model, the non-green area (i.e., not leaf such as branches and other noises), the normalized green value of which (G/(R + G + B)) was less than 0.4, was cut. All points constituting the 3D model were converted into voxel coordinates (voxel-based 3D model), in which each X, Y and Z value of point-cloud data was rounded-off to the nearest integer value, allowing it to efficiently calculate structural parameters [12,13,15,18,19]. The size of a voxel was set to about between 0.03 and 0.2 cm, determined per the plant scale. Smaller voxel sizes were given to smaller plants. Voxels corresponding to coordinates converted from points within the data were assigned an attribute value of 1, and the space without points was given an attribute value of 0 [39]. 

#### 2.3.2. Generation of Top-View Binary 2D Image from Voxel-Based 3D Model 

Each point in the voxel-based 3D model was projected onto the plane above the model, and the binary 2D images of the top view could be obtained. Pixel values in the binary image were the same as the corresponding voxels. In the projected images, regions of leaves included small holes caused by lack of points. The holes were filled using an algorithm based on morphological reconstruction, in which a hole was filled if the pixel values around the hole were not zero [40]. 

#### 2.3.3. Generation of Seed Regions for 3D Leaf Segmentation

**Figure 1:**
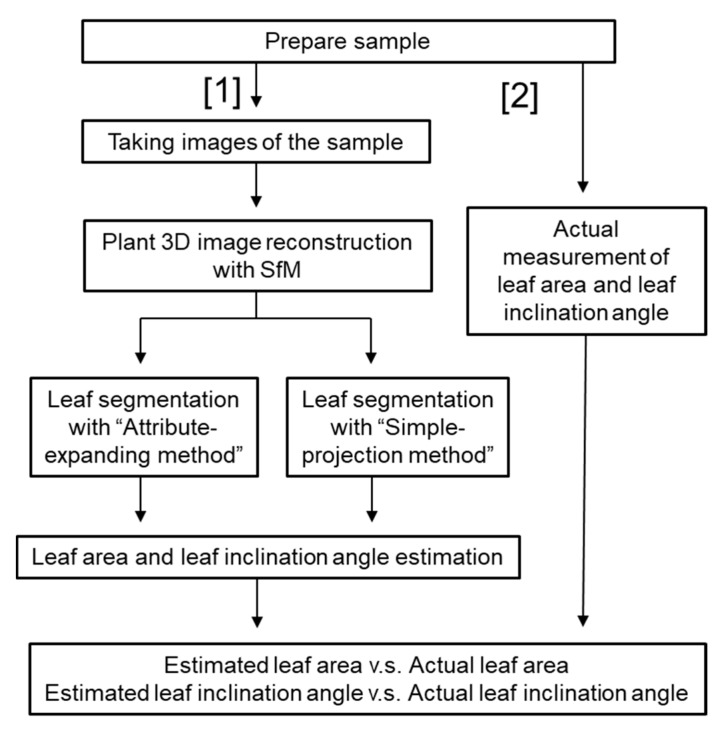
Flow chart of the process from 3D reconstruction, leaf segmentation to estimation of structural parameters.

**Figure 2:**
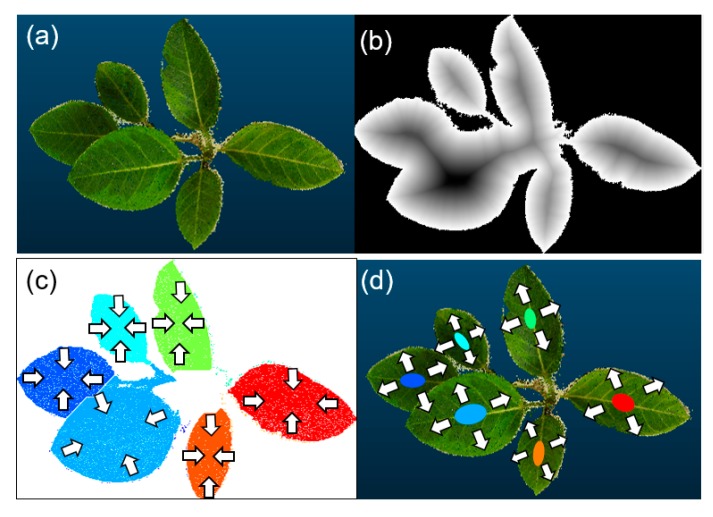
Top-view 2D images of a plant generating initial seed regions for 3D leaf segmentation: (**a**) top-view image of a 3D plant point-cloud image; (**b**) grayscale image via distance transform—the contrast represents the distance from the nearest edges; (**c**) an image after initial segmentation by the watershed algorithm—colors represent each leaf and arrays indicate the directions of shrinking to create seed regions; and (**d**) an image representing seed regions for the 3D leaf segmentation—arrays show the directions for expanding each region in the 3D images.

The flow chart of the experimental process is shown in Figure 1. Next, the method for leaf segmentation is explained. In the top-view 2D binary images, the distance transform of [41] was implemented, for which the distance between each background pixel (pixel value: 0) and the nonzero pixel (leaf or plant organs) nearest the background was calculated [42]. The border of the background and nonzero pixel consisted of leaf edges. Figure 2a,b represent the top-view image of a 3D point cloud and the grayscale image via distance transform, respectively. In the grayscale image (Figure 2b), the contrast represents the distance from the nearest leaf edges. Areas closer to the leaf edges are illustrated by brighter colors. Then, the watershed algorithm [43] was conducted to the distance transformed image as the initial leaf segmentation process. Afterwards, the segment numbers (i.e., leaf number) were labeled at each pixel corresponding to segmented leaves in the 2D image. The initial leaf segmentation result is shown in Figure 2c. Regions of zero pixels had no leaf numbers. The initial 2D leaf segmentation was insufficient because the occluded regions and boundaries between each leaf were not correctly segmented. Afterwards, the scale of the image after the initial segmentation was reduced to 1/10 around its centroid (arrows in Figure 2c shows the directions of reduction). The 1/10 parts (i.e., seed region) were projected onto voxels in the 3D model (Figure 2d), and the voxels corresponding to the seed regions were assigned to the leaf number.

#### 2.3.4. Automatic Leaf Segmentation by Expanding the Seed Region

Next, leaf numbers were assigned to the nonzero voxels neighboring the seed regions. Then, the leaf number was also assigned to the nonzero voxels neighboring the voxels per leaf number. By repeating this process, a leaf number was assigned to each nonzero voxel, and each seed region was expanded three-dimensionally, as shown in Figure 2d. This process was repeated until leaf numbers were assigned to all nonzero voxels and leaf segmentation processes were finished. This method has not been introduced before and we call it the “attribute-expanding method” in this paper. 

### 2.4. Automatic Leaf Area and Leaf Inclination Angle Estimation from Segmented Leaves

Leaf area and leaf inclination angle were estimated from segmented leaves. In the leaf area calculation, the voxel sizes of the X and Y axes were not changed, but the voxel size of the Z axis was changed to 3.0 to reduce the influence of noises around leaf surfaces in a vertical direction, which results in overestimation of leaf area. The leaf area was calculated by multiplying the total number of voxels within a leaf and an area of a horizontal face of a voxel. 

For leaf inclination angle estimation, Hosoi et al. [44] fitted a plane onto one leaf, and the inclination angle was calculated from the normal vector. Based on this study, the inclination angle was calculated for each leaf by fitting points around the centroid point of each segmented leaf. 

### 2.5. Evaluation of the Accuracy of Automatic Leaf Segmentation and Leaf Area and Leaf Inclination Angle Estimation

To evaluate the accuracy of automatic leaf segmentation, leaf area estimates after manual segmentation from the 3D images and ones from the present automatic leaf segmentation were compared (n = 61). Success rate was defined also for evaluating the accuracy of the present segmentation. Success rate was the rate of the number of the correctly segmented leaves to the total number of all leaves within a plant. If the percentage error of the automatic leaf area estimation was less than 10%, it was regarded as a correct segmentation, that is, high enough to be practical [28].

Next, performance of segmentation methods to leaves overlapped in the top view was compared to the present segmentation method and the 2D image-based method. The latter is a method simply projecting the initial segmentation result (e.g., Figure 2c) to the 3D plant model (i.e., simple-projection method). We prepared 10 sets of two leaves which were overlapped in the top view. Then, the accuracy of leaf area estimation after segmentation was compared to the results of the two methods. Then, to evaluate the accuracy of the voxel-based leaf area estimation, the estimated leaf area and actual leaf area were compared (n = 30). For obtaining actual leaf area, JPEG images of leaves were taken, and the areas were determined by multiplying the number of pixels and the area per pixel. When taking the JPEG images, leaves were pressed between a transparent board and white board to flatten them. 

The actual value of the leaf inclination angle measured by an inclinometer and its estimated and actual values were compared (n = 30) to evaluate the error of leaf area estimation.

## 3. Results

### 3.1. Leaf Segmentation

**Figure 3:**
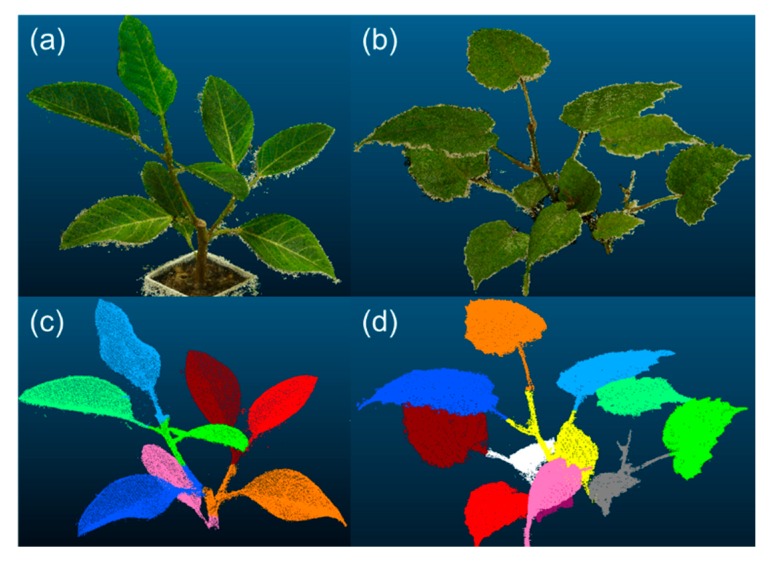
Segmentation results of plants: plants in image (**a**) (Council tree) and (**b**) (Kangaroo vine) have 8 and 11 leaves, respectively; images (**a**,**b**) represent 3D point-cloud images of the target plants; images (**c**,**d**) show the results of segmentation of images (**a**,**b**), respectively.

**Figure 4:**
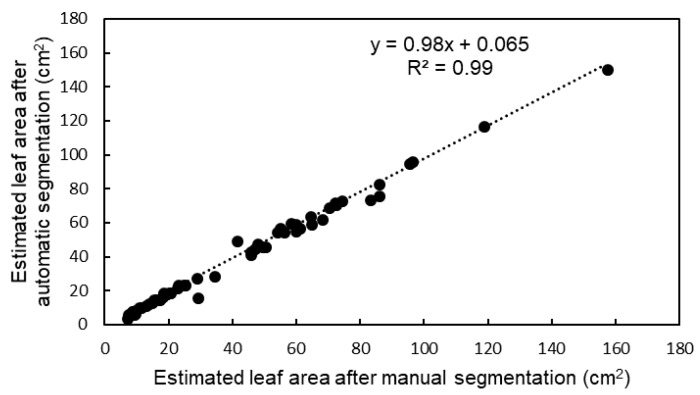
Relationship between leaf area estimates after manual segmentation and those after automatic leaf segmentation.

Figure 3 illustrates examples of segmentation results. Plants in images (a) and (b) had 8 and 11 leaves, respectively. The attribute-expanding method enabled the accurate segmentation of the plant with such structures. Table 1 represents the accuracy of leaf area estimation and leaf segmentation. As shown in the table, for all segmented leaves, the success rate was 86.9%. Absolute leaf area estimation error showed an increasing tendency with increasing leaf area. Figure 4 shows the relationship between leaf area estimates after manual segmentation and those after automatic leaf segmentation. This indicates a high correlation of *R*^2^ = 0.99 for the leaf area with the present automatic method, compared to that with manual segmentation. Its root-mean-square error (RMSE) was 3.23 cm^2^. Leaf area estimates by the simple-projection method were also compared to ones after manual segmentation, offering an RMSE of 8.26 cm^2^, much higher than the present automatic segmentation. 

### 3.2. Leaf Area and Leaf Inclination Angle Estimation from Segmented Leaves

**Figure 5:**
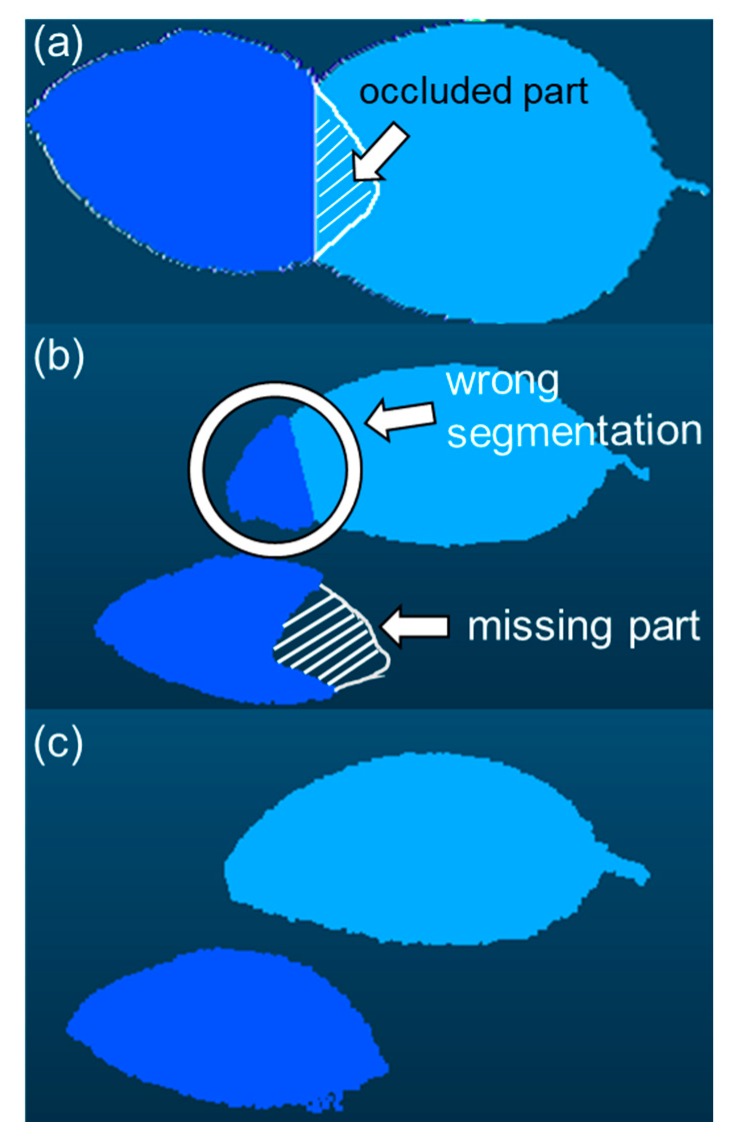
Example of segmentation for overlapped leaves: image (**a**) is a 2D image after initial segmentation, and image (**b**,**c**) show the images after segmentation via the simple-projection and attribute-expansion methods, respectively.

Figure 5 depicts an example of segmentation for overlapping leaves. Figure 5a represents a 2D image after initial segmentation (explained in Section 2.3.3). After obtaining the image, leaf segmentation was conducted via the simple-projection method (Figure 5b) and the attribute-expanding method (Figure 5c). In Figure 5b, an attribute value was not assigned to the part of the lower leaf occluded by the above leaf in the top-view image (Figure 5a). Consequently, the occluded part was missed in the simple-projection method. Moreover, in Figure 5b, the tip part above the leaf and the region around the leaf boundary was wrongly segmented. The segmentation error problem was later solved during the attribute-expanding method, as shown in Figure 5c.

During the segmentation of 10 sets of overlapped leaves, the leaf area estimation error (mean absolute percent error: MAPE) through the simple-projection method (Figure 5b) was 17.4% ± 7.1%, whereas the error with the attribute-expanding method (Figure 5c) was 1.4% ± 2.0%, significantly smaller (*p* < 0.01). 

**Figure 6:**
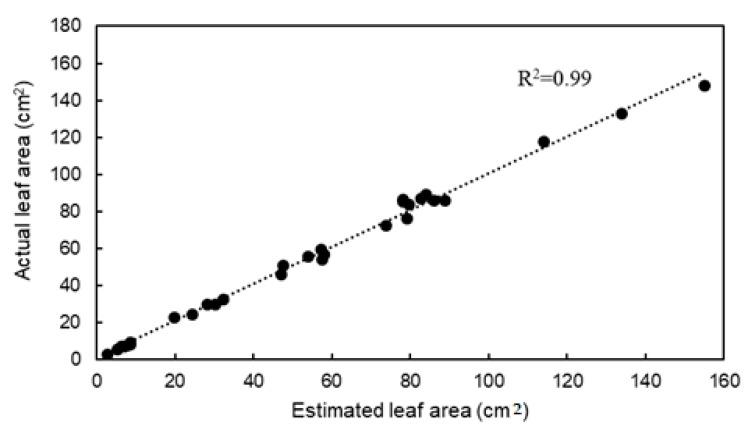
Relationship between estimated leaf area based on number of voxels and actual leaf area.

Figure 6 shows the relationship between estimated leaf area based on number of voxels in the 3D models and the measured leaf area based on JPEG images. The coefficient of determination was 0.99, and the RMSE and the MAPE were 3.21 cm^2^ and 4.14%, respectively.

In the leaf inclination angle estimation, the RMSE and absolute error of each segmented leaf was 2.68° and 1.92°, respectively. The estimation accuracy was independent of the inclination angle of the target leaf.

## 4. Discussion

### 4.1. Leaf Segmentation

In the top-view 2D images of plants, some regions of leaves were occluded because of leaf overlapping, and boundaries between leaves were not clear. Thus, in the simple-projection method, unsegmented missing parts and wrong segmentation in the leaf borders were unavoidable, as shown in Figure 5b. Similar problems could exist in other 2D image-based segmentations, such as graph-cut or active contour models [35,36]. On the other hand, the missing parts and the wrong segmentation on the leaves were not observed in the leaves segmented with the present method. It resulted in more accurate leaf segmentation and leaf area estimation. Moreover, recent methods for automatic mesh-based 3D reconstruction have started to be considered [45,46], however, it is still very difficult to conduct the reconstruction fully automatically. Unlike the mesh-based 3D models, the construction of voxel-based 3D models can be done automatically; also, on this point, our voxel-based segmentation method is advantageous.

Unlike simple projections, in the attribute-expanding method, distance transform and the watershed algorithm were used not for segmentation of all leaf regions but for making seed regions around each leaf centroid in the 3D models. Furthermore, the seed regions were projected onto the 3D models, and each leaf region was three-dimensionally expanded from the projected seed regions, assigning the leaf number to the voxels of leaves. The region expansion stopped at the leaf edges and did not progress to other leaves because each leaf edge within a leaf was apart from the edges of other leaves in the 3D coordinates. Hence, whereas the leaf edges could not be segmented correctly in the simple-projection method because of leaf overlapping in the top-view 2D image, leaf edges could be segmented accurately in the present segmentation method, as shown in Figure 5c. Moreover, such 3D region expansion also allowed assignment of the leaf number to the region occluded by the top-view 2D image, resulting in accurate leaf segmentation without missing parts (Figure 5c). When leaves and branches cannot be distinguished, the segmentation accuracy decreases. Using the normalized green value (G/(R + G + B)), branches were automatically cut down, however, a few branches remained, which resulted in the segmentation error. By optimizing the normalized green value at each sample depending on the color of the target leaf and branches and/or light condition, the error will be lower. The optimization of the value will widen its application. 

### 4.2. Voxel-Based Leaf Area Calculation and Leaf Inclination Angle Estimation

The MAPE of the leaf area estimation with the mesh-based methods ranged from 2.41% to 10.3% [16,35,47,48] in previous studies, whereas MAPE in the present method was 4.14%, meaning the leaf area estimation accuracy in this method was high enough. In the mesh-based 3D model, a mesh is composed of a target point and the adjacent two points. The meshes had to be constructed with whole-leaf surfaces to retrieve leaf areas and inclination angles of all leaves. However, mesh formation through the whole-leaf surface was difficult in the complicated 3D point configuration often observed in plant 3D models, resulting in holes (i.e., regions where mesh construction failed) on the surfaces. To fill the holes, manual interpolation by additional mesh surfaces was needed. However, the present voxel-based calculation estimated leaf area by simply counting the number of voxels and converting them to a surface area without any manual processes needed by the mesh-based calculation. This allowed fully automatic leaf area calculation. Moreover, because points in the present 3D models were constructed from SfM and distributed thoroughly on each leaf with high density, no holes were on leaves of the voxel models. This led to accurate leaf area calculation.

The accuracy of leaf inclination angle estimation in this study was as high as the previous study (RMSE in the previous study: 4.3° [44]). Thanks to the automatically segmented leaf, a plane fitting to each leaf for the angle estimation could be automatically conducted to each leaf, such that the inclination angle of each leaf could be estimated automatically, whereas the mesh-based method could estimate the inclination angle from the normal mesh vector. Thus, manual operation was necessary to fill the holes of leaf surfaces. 

As described above, the present voxel-based leaf area and leaf inclination angle calculations were equivalent to or greater than the mesh-based methods in terms of accuracy and fully automatic calculation. 

## 5. Conclusions

In this study, plant 3D point-cloud images were constructed using SfM, and leaves in the 3D models were segmented automatically; then, leaf area and leaf inclination angle were estimated automatically. First, initial segmentation was conducted based on the top-view images of plants using distance transform and the watershed algorithm. Next, the images of leaves after the initial segmentation were reduced by 90%, and the seed regions for each leaf were produced. Then, the seed regions were projected onto the voxelized 3D models. Afterwards, each region with a different leaf number was expanded on the 3D model. Finally, each leaf in the 3D models was segmented. From the segmented leaves, each leaf area and leaf inclination angle was estimated using a voxel-based 3D image processing method. As a result, more accurate and automatic leaf segmentation was realized compared to previous studies. In all segmented leaves, the success rate was 86.9%. Leaf area estimates after manual segmentation and those after automatic leaf segmentation indicated a high correlation of *R*^2^ = 0.99 for the leaf area with the present automatic method. Its RMSE was 3.23 cm^2^. In the leaf inclination angle estimation, the absolute error of each segmented leaf was 1.92°. Thus, a series of processes from automatic leaf segmentation to structural parameters estimation was conducted automatically and accurately. The present method offers efficiently useful information for appropriate plant breeding and growth management. As a next step, this method should be applied to various kinds of plants to ensure its potential to be used widely. 

## Figures and Tables

**Table 1:** Accuracy of leaves are estimations and leaf segmentation of each plant; success rate represents the percentage of leaves correctly segmented with more than 90% leaf area estimation accuracy.

	Average Leaf Number	Leaf Area (cm^2^)	Absolute Leaf Area Estimation Error (cm^2^)	Success Rate (%)
Dwarf schefflera	5	8.06	0.06	100
Kangaroo vine	11	13.74	0.67	82
Pothos	4	20.67	0.29	100
Hydrangea	4	41.48	1.66	100
Council tree	5.3	59.68	3.44	75
Dwarf schefflera	5	73.48	2.35	90
All sample	5.7	36.2	1.73	86.9
